# Sound Levels Forecasting in an Acoustic Sensor Network Using a Deep Neural Network

**DOI:** 10.3390/s20030903

**Published:** 2020-02-07

**Authors:** Juan M. Navarro, Raquel Martínez-España, Andrés Bueno-Crespo, Ramón Martínez, José M. Cecilia

**Affiliations:** 1 Escuela Politécnica. Universidad Católica de Murcia (UCAM), Campus de los Jeronimos, 30107 Guadalupe, Spain; 2 Computer and Systems Department (DISCA). Universitat Politècnica de València (UPV), Camino de Vera, s/n, 46022 Valencia, Spain

**Keywords:** acoustics, wireless sensor networks, smart cities, deep learning, long short-term memory, temporal forecast

## Abstract

Wireless acoustic sensor networks are nowadays an essential tool for noise pollution monitoring and managing in cities. The increased computing capacity of the nodes that create the network is allowing the addition of processing algorithms and artificial intelligence that provide more information about the sound sources and environment, e.g., detect sound events or calculate loudness. Several models to predict sound pressure levels in cities are available, mainly road, railway and aerial traffic noise. However, these models are mostly based in auxiliary data, e.g., vehicles flow or street geometry, and predict equivalent levels for a temporal long-term. Therefore, forecasting of temporal short-term sound levels could be a helpful tool for urban planners and managers. In this work, a Long Short-Term Memory (LSTM) deep neural network technique is proposed to model temporal behavior of sound levels at a certain location, both sound pressure level and loudness level, in order to predict near-time future values. The proposed technique can be trained for and integrated in every node of a sensor network to provide novel functionalities, e.g., a method of early warning against noise pollution and of backup in case of node or network malfunction. To validate this approach, one-minute period equivalent sound levels, captured in a two-month measurement campaign by a node of a deployed network of acoustic sensors, have been used to train it and to obtain different forecasting models. Assessments of the developed LSTM models and Auto regressive integrated moving average models were performed to predict sound levels for several time periods, from 1 to 60 min. Comparison of the results show that the LSTM models outperform the statistics-based models. In general, the LSTM models achieve a prediction of values with a mean square error less than 4.3 dB for sound pressure level and less than 2 phons for loudness. Moreover, the goodness of fit of the LSTM models and the behavior pattern of the data in terms of prediction of sound levels are satisfactory.

## 1. Introduction

Noise pollution is one of the main environmental concerns of modern cities because of its effects on the quality of life, health and livability of cities. The European Commission adopted the European Noise Directive (END) [1], which focuses on the monitoring of environmental noise by generating noise maps of the main population centers and elaborating action plans [2,3]. Noise measurements in urban areas are typically carried out by designated officers that collect data at a few accessible spots, where sound level meters are installed during short time intervals. Collected noise data is often input into a model that attempts to predict noise levels for a temporal long-term throughout the landscape to be evaluated. As a result, noise maps are generated using sound sources and propagation models leveraging geographic information systems to improve the accuracy and quality of the results [4,5]. Specifically, road [6,7,8,9,10], railway [11,12,13] and aerial [14,15] traffic models are used, among others. However, according to Maisonneuve [16], this approach presents several limitations since noise maps are actually generated from synthetic data. Even though these models allow to gain a first insight into the noise pollution problem, they are mainly focused on long-term acoustic parameters prediction and require auxiliary data such as source definition, traffic flow, street geometry, day period, urban topology, etc.

Wireless Acoustic Sensor Networks (WASN) [17,18] are becoming an indispensable tool for monitoring and assessment of short-term noise levels. WASN are a balanced technology regarding the characteristics of cost, scalability, flexibility, reliability and accuracy [19]. Such networks are supported by recent advances in low-power wireless communications technology as well as the integration of several functionalities in electronic devices, including sensing, communication and processing, even allowing the implementation of neural networks in the nodes [20]. They are being extensively used in smart city applications in recent years. This trend has led to intensive deployments in numerous cities such as New York [21], Barcelona [22] or Monza [23]. WASN can be deployed over an area of interest to operate continuously by creating a real-time monitoring system, which collects historical data related to the sound environment over longer periods of time, operating unattended and requiring human intervention only for network installation, maintenance and removal. This data is transmitted to a central sink node, then could be stored and subsequently be used, for instance, to dynamically update noise maps [24]. Indeed, all these information acquired by WASN can be analyzed to obtain useful information for the city [25]. Moreover, it is very interesting and relevant to predict the short-term behavior of the acoustic parameters that evaluate the sound environment. For instance, it allows the ability to detect behavior patterns depending on different times of day and, furthermore, in the event of failure or error in sending information from a sensor, this information can be estimated with precision. In addition, by being able to know these unique level values several days in advance, preventive measures could be taken if necessary to avoid the population from being exposed to risk levels. Therefore, in this work, a novel approach based on deep neural networks is introduced to forecast the near-time short-term sound level values using only historical sound level data from the location of study. In this way, the approach that is presented in this paper can be applied to every node of the sensor network, where the inputs of the model are the past and actual sound level values and the outputs are the future values.

To achieve this objective, in this paper the use of the Long Short-Term Memory (LSTM) deep neural network technique is proposed to model the behavior pattern of the acoustic parameters which has demonstrated very good results in prediction of time series [26,27]. Sound sources, specifically those concerning a sound environment in this work, can be considered as time variant functions, i.e., time series, both the audio signal and the corresponding calculated parameters. Time series data analysis has been actively researched for decades and is considered one of the ten most difficult problems in data mining due to its unique properties. In this work, the capability of LSTM networks to estimate short-term future values of sound levels in a certain location using historical data is explored. In particular, several models are obtained by training the LSTM networks with sound pressure level and loudness level values captured by a node of a WASN. Comparison with ARIMA technique results together with some experiments are presented to evaluate the proposed approach.

The paper is structured as follows. After this introduction, Section 2 presents a review of related work and the difference with the proposed approach. Then, Section 3 describes the deployed sensor network, designed LSTM networks and the collection and pre-processing of the data-set handled to train and evaluate them. In Section 4 different results obtained from the experiments to evaluate the implemented LSTM networks are shown and discussed. Finally, Section 5 presents the general conclusions of this study and proposes future work.

## 2. Related Work

A significant amount of information generated by sound sources is carried by acoustic signals, and this information can be used to describe and understand human and social activities. Sound signal acquired by acoustic sensors can be processed in two ways: (i) capturing and processing the audio signal (e.g., event detection [28,29], classification of sound sources [30,31], sound source location [32], etc.) and (ii) calculating values of acoustic parameters from the captured audio signal (e.g., sound pressure level [33], loudness [34], etc.) that are the data collected to generate sound maps.

Several works have been developed in applying artificial neural networks to estimate sound source features and/or acoustic parameters values in a certain location for a given period of time, using data obtained through WASN or other information data base. In what follows we introduce differences between the proposed work and these previous works. Regarding audio signal processing, in publications [35,36] a WASN is proposed to monitor and analyze urban noise pollution, deploying a network of sensors to measure sound pressure level and using convolutional neural networks to classify sound sources from captured audio. In other work, Socoró et al. [37] introduced an anomalous noise event detector to remove sound frames unrelated to road traffic sound sources to provide more reliable data captured by a WASN. In [38], a convolutional recurrent neural network in a dilated spiral is used as a classifier fed by the energy recording feature in the mel band for the detection of sound events. Regarding to parameters calculation, some published papers introduce neural networks to estimate advanced acoustic parameters values. Yu and Kang [39] explored the feasibility of using machine learning models to predict the sound landscape quality in urban open spaces by correlating various physical, behavioral, social, demographic and psychological factors. In [40], a convolutional neural network was implemented to estimate the psycho-acoustic annoyance Zwicker’s model from an input audio signal. In contrast with these related works, in our research a neural network approach is used to predict future time values of acoustic parameters instead of estimating current time values.

There are some studies that apply neural networks to create a prediction model in order to estimate sound pressure levels emitted by sound sources across a spatial domain but using also geospatial and description information as input parameters. Specifically in [41], a multi-layer perceptron neural network model trained with the Levenberg–Marquardt algorithm was used to predict the equivalent sound level from road traffic noise. In another publication [42], a system proposition is presented that uses an ensemble of machine learning techniques to estimate both environmental sound levels and uncertainty in model predictions by taking geospatial data as input. In addition to making use of auxiliary information, these neural network-based models predict long-term values and do not take into account the temporal composition of the short-term sound environment. An attempt to predict the temporal component of traffic noise levels is presented in [43] through the use of back-propagation neural networks, however it only estimates index values describing temporal variability and impulsiveness in addition to using auxiliary data as input. Although noise sources are mainly non-stationary, statistical techniques such as AutoRegressive Integrated Moving Average (ARIMA) [44] have been also used in the literature to model traffic noise pollution.

Finally, it is worth highlighting that there are several works in the literature that predict other pollution factors through deep neural networks, considering the data of these variables as time series. Specifically, the most common pollution problem studied is air pollution, particulate matter and carbon monoxide concentrations among others [45,46]. However, the use of deep learning models such as LSTM require an optimized configuration and settings for each type of problem, as it is carried out in Section 3.5, considering the inputs and its behavior in time.

## 3. Materials and Methods

### 3.1. Wireless Acoustic Sensor Network

In this work, data captured from a node of a deployed WASN was used to train and validate the designed neural network prediction models. This WASN is a scalable and extensible system used to monitor sound levels in a certain environment. This is a static and homogeneous WASN allowing continuous monitoring indoors and outdoors. This network was composed of ten acoustic nodes deployed in the campus of the Catholic University of Murcia. In this WASN, each acoustic node [47] collected and processed the audio signal and after that, it calculated and sent data every minute to the sink node. The low-cost acoustic node design included two main parts: the audio acquisition system and the processing core. The former consisted of an array of the four-microphones of a Sony PlayStation Eye camera. Regarding the processing core, a Raspberry Pi 3 Model B computer [48] was selected for the processing, acquisition and publishing stages. Although a node is able to compute results related to diverse acoustic parameters, see [47] for details, this research is focused on the equivalent sound pressure level ($L_{p}$) and loudness level (*N*) values [49] in a one-minute period. A sink node plays the additional role of transmitting the data to an Internet of Things (IoT) platform to store and to perform analysis of the overall data. The audio signal was not stored nor transmitted from the node to keep public privacy. Concerning the network design, acoustic nodes transmit data via Wi-Fi technology using two communications protocols: TCP for communication between nodes and HTTP for communication between the sink node and the IoT platform. Further in-depth control and maintenance of the deployed nodes was provided via a virtual private network that provides a method for remote Secure SHell (SSH) access to each node. The virtual private network also enhances the wireless transmission security of the sensor as all data and control traffic was routed through this secure network.

Specifically for this research, a data-set with these acoustic parameters, $L_{p}$ and *N*, was built, as it is explained in detail in the following section.

### 3.2. Acoustic Data-Set

In this research, the acoustic data acquired on a continuous basis with a temporal period, i.e., a time step of 1 min by a node of the described WASN in the previous section was used to train a LSTM network. This data-set was collected from the beginning of October to the end of November 2019 and it contains quantitative and temporal data related to two acoustic parameters: the equivalent sound pressure level in decibels (dB) and loudness level in phons in one-minute of integration time. The selected node was located in-door in an open-office room where lecturers and researchers work. Working days are mainly from Monday to Friday but Saturday is also open. This data-set is representative of a random noise, of which the main sound sources are speech and human activities. This long-period study can help to analyze and predict the temporal behavior pattern of this type of soundscape.

From the principal data-set, a total of ten data-sets have been generated, five for each parameter, computing a temporal average of the data for the following periods: 1, 5, 15, 30 and 60 min. The following average has been used for time intervals:

(1) $$X=10log\left(\frac{1}{n}\sum_{i=1}^{n}10^{\frac{X_{i}}{10}}\right),$$

where *X* can be either $L_{p}$ or *N*, and $X_{i}$ corresponds respectively to the equivalent sound pressure level ($L_{p_{i}}$) and loudness level ($N_{i}$) for each time step *i*. For example, the data-set denoted as noise15 in Table 1 indicates that the 1-min values have been averaged over 15 min, generating one value for $L_{p}$ and other for *N*. A description of the quantity of samples used for each data-set can be seen in Table 1. The number of samples in each data-set corresponds to approximately 50 days.

### 3.3. Deep Learning: Long Short-Term Memory

**Figure 1:**
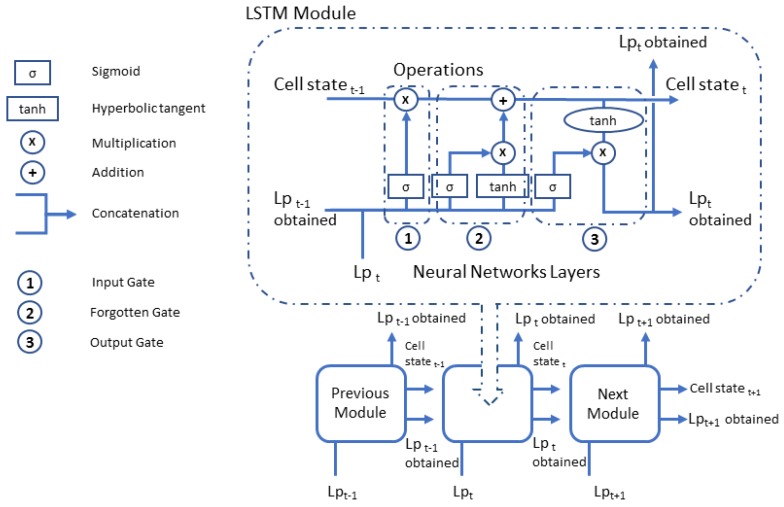
General scheme of an Long Short-Term Memory neural networks (LSTM) for $L_{p}$. The interaction between LSTM modules can be observed, as well as the three types of gates that make up an LSTM module.

A Recurrent Neural Network (RNN) in very powerful for everything that has to do with sequence analysis, such as text, sound or video analysis. The main feature of an RNN is that information can persist by looping into the network diagram, so they can basically “remember” previous states and use this information to decide what will be next. This feature makes them very suitable for managing time series. However, a conventional RNN presents problems in training because retro-propagated gradients tend to grow enormously or fade over time because the gradient depends not only on the present error but also on past errors. The accumulation of errors makes it difficult to memorize long-term dependencies. These problems are solved by the Long Short-Term Memory neural networks (LSTM), for which it incorporates a series of steps to decide which information will be stored and which erased. The LSTM networks are composed of LSTM modules which are a special type of recurrent neural network described in 1997 by Hochreiter and Schmidhuber [50]. The LSTM module contains three internal gates, known as input, forgotten and output (as can be seen in more detail in the Figure 1), consisting basically of a sigmoid layer and a multiplication operation, and in the case of the forgetting door, it also incorporates a hyperbolic tangent layer. These gates allow to remove or add information to the cell state, which is a connection that transfers information from one LSTM module to the next. The input gates controls when new information can enter memory. Forgotten gates control when a piece of information is forgotten, allowing the cell state to discriminate between important and superfluous data, leaving room for new data, for this, a hyperbolic tangent layer is added which is combined with the sigmoid layer. Output gate controls when used in the result of memories stored in the cell state. The cell state has a weighting optimization mechanism based on the resulting network output error, which controls each gate. The output and the cell state value generated by the LSTM module are transferred to the next LSTM module. Figure 1 shows the gates and operations of an LSTM module graphically for $L_{p}$ (for *N* it would be the same scheme), and in which it can be observed that the input for a unit, is the output of the previous one. This way, each LSTM module transmits to the next one its prediction that together with the current input of the module, generate the output that is sent as input to the next LSTM module.

The network proposed in this work is univariate, that is, it takes a single input variable and obtains a single output variable, given that the objective of the work is to predict both the L_p_ sound levels and the loudness *N*. Thus, for the prediction of each one of these values, a different LSTM model will be made for each data-set.

### 3.4. Statistical Approach: Auto Regressive Integrated Moving Average

Classical approach to predict time-series is based in statistics. The Auto Regressive Integrated Moving Average technique [51] is a statistical model that uses variations and regressions of statistical data in order to find patterns for a prediction into the future. It has been also applied to sound level parameters prediction [44], as it has been introduced in Section 2. ARIMA is a dynamic time series model, i.e., future estimates are explained by past data rather than independent variables. This model was developed in the late 1960s. Box and Jenkins (1976) systematized it [52]. An ARIMA model is characterized by 3 terms: (p,d,q,) where, *p* is the order of the Auto Regressive (AR) term, *q* is the order of the Moving Average (MA) term and *d* is the number of differences needed to make the time series stationary. In this work, an ARIMA model has been created using the same data-set described in Section 3.2 to compare with quality metrics of the proposed LSTM models.

### 3.5. Experiment Configuration

The viability and suitability of the proposed LSTM technique is assessed using two types of experiments. On the one hand, an experiment was executed using 80% of the data-set to train the model and 20% to test it. This experiment was applied to the five data-sets (different time intervals) described in Section 3.2, for each acoustic parameter. In addition, to validate the LSTM model, we performed a comparison with the Auto Regressive Integrated Moving Average (ARIMA) technique [51]. On the other hand, to analyze the robustness and adaptability of the proposed LSTM model, we performed several types of validation for the 30 and 60 min data-sets, which are the best results obtained globally. Specifically on the proposed LSTM model; comparisons will be made using the validations of 60%, 70% and 80% to train and 40%, 30% and 20% to test respectively. Thus, depending on the results, the response capacity of the model presented can be analyzed in the absence of training data.

For the ARIMA model, used in the comparison, the parameter (p,d,q) used for the for the estimation of the acoustic parameter $L_{p}$ were (1,1,14) and for the acoustic parameter *N* were (1,1,10). In the LSTM model proposed in this paper, the optimal parameters that have been chosen, after a previous adjustment carried out to obtain the optimum parameters, are shown in the Table 2. For the number of neurons, intervals are shown depending on the acoustic parameter.

The quality evaluation of the model proposed is performed by measuring the goodness of the prediction by the following metrics:

- the Root Mean Square Error (RMSE)
- the Mean Absolute Error (MAE)
- the Pearson Correlation Coefficient (PCC)
- Determination Coefficient ($R^{2}$)

Experiments were been carried out in a GPU-based platform. This platform was composed of an Intel(R) Xeon(R) CPU E5-2640 v4 @ 2.40GHz, 128 GB of RAM, 1 TB SSD Hard Disk and a NVIDIA GeForce GTX 780 GPU (Kepler).

## 4. Results and Discussion

In this section, the behavior of the LSTM model proposed for the prediction of the sound pressure level and loudness values is discussed and analyzed. The evaluation and analysis is detailed in two subsections. First, a comparison with a technique to predict the time series of ARIMA was made by performing an experiment with 80% of the data-set to train and 20% to test. Then, to validate the robustness of the proposed LSTM technique, several validations increasing the test percentage and reducing the train percentage were performed. It should be noted that the predictions were estimated for the values $L_{p}$ and *N*, therefore for each of these values a different model was made.

### 4.1. Comparing the LSTM Model with the ARIMA Model

This section presents the results obtained by the LSTM models for the prediction of the parameters $L_{p}$ and *N* for the different data-sets described in Section 1. In addition, LSTM models are compared with the ARIMA technique models for both parameters to validate the results. The validation carried out for both LSTM and ARIMA models was using 80% of the data-sets to train and 20% to test. The number of days is equivalent to about 40 days for training and about 10 consecutive days of prediction for testing.

Table 3 shows the values of RMSE, MAE, PCC and $R^{2}$ for each of the data-set of $L_{p}$ parameter for the LSTM and ARIMA models. For the LSTM models, the calculated metrics are very satisfactory in general, obtaining a RMSE lower than 4.3 dB for $L_{p}$ in all the data-sets. Regarding to the fit of the model, $R^{2}$, the better is this fit the greater the temporal amplitude of the interval is. This may be caused by the smoothing obtained by the averaging of punctual noise peaks. The best fit of the model, 0.75, is obtained for $L_{p}$ when the prediction period is 60 min. With respect to ARIMA models, the RMSE values increase considerably, which indicates that the ARIMA technique is not adequate for estimating the behavior of the $L_{p}$ parameter in short-term intervals. For all data-sets the ARIMA model fit is very low and the errors much higher than for the LSTM model. It must be taken into account that ARIMA may need more days of training to be able to reduce the error and improve the fit of the predicted time series. This is one of the advantages of the LSTM technique.

Table 4 shows the values of RMSE, MAE, PCC and $R^{2}$ for each of the data-set of *N* parameter for the LSTM and ARIMA models. For the LSTM models, the calculated metrics are very satisfactory in general, obtaining a RMSE lower than 2 phons for *N* in all the data-sets. Particularly, metrics show that the RMSE of *N* is similar for all time intervals. In addition, the value of adjustment of the model, $R^{2}$, of *N* is very similar in all the cases, which indicates that it is less affected by the time interval considered to predict sound levels. For ARIMA models, the behavior and results for predicting the *N* parameter is similar to the $L_{p}$ parameter. In this case, the error does not increase as significantly as for the $L_{p}$ parameter. However, the error is always more than double that obtained by the LSTM technique. Moreover, as far as the model’s adjustment is concerned, the result is not at all satisfactory. This indicates that the ARIMA models are not able to adapt to the non-stationary behavior of the sound level parameters in short-term intervals.

**Figure 2:**
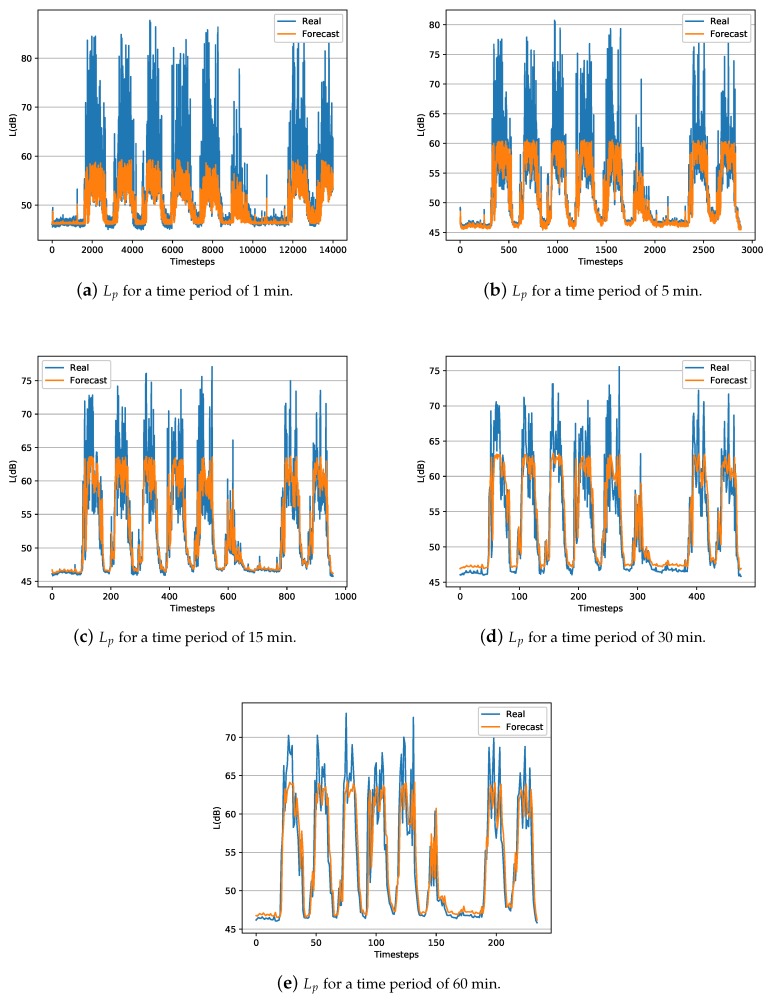
Representation of captured and estimated LSTM data during approximately ten days test-interval (20% of the data) of $L_{p}$.

**Figure 3:**
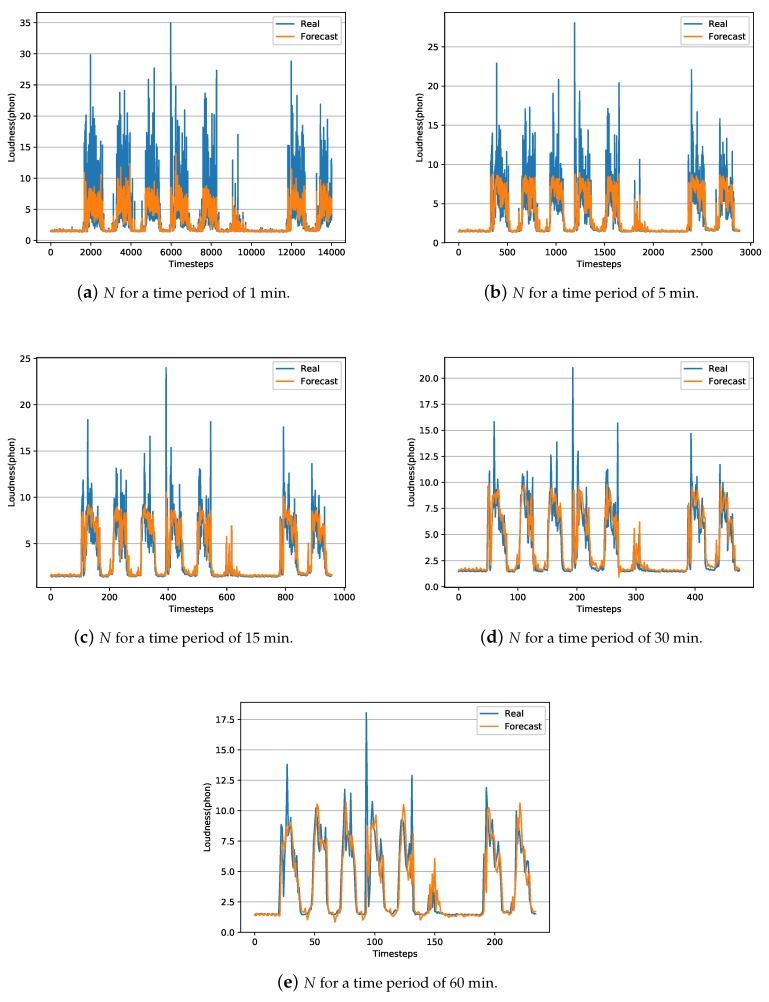
Representation of captured and estimated LSTM data during approximately ten days test-interval (20% of the data) of *N*.

In summary, results show that the LSTM technique outperforms the ARIMA technique for creating temporal short-term models and predicts the behavior of the $L_{p}$ and *N* parameters. One aspect to consider about the obtained LSTM models is the difference between the RMSE and MAE values for both *N* and $L_{p}$ levels. The MAE value is almost double the RMSE value, indicating that there are outliers in the data [53]. These outliers data are usually reflected by the peaks. In this case, the outliers can be observed in Figure 2 and Figure 3, for both *N* and $L_{p}$ levels, in the eventually impulsive sound events that occur throughout the day.

Figure 2 and Figure 3 represent a temporal graph for a ten days interval of the captured data, i.e., real data from the test-subset, along with the estimated data using the obtained LSTM models for both *N* and $L_{p}$. The test-subset begins on Sunday and ends on Tuesday of the following week. Therefore, it can be observed that the minimum noise level on Sunday because the open-office room where the data has been collected is closed. However, the acoustic level increases over the next five working days on the day-period and decreases on the night-period. On Saturday, the activity of people in the office is reduced, thus the noise level is quieter than a regular working day. Then, the time sequence starts again with a Sunday having the lowest noise levels. In general, the model obtained by the LSTM technique, as a pattern of sound level behavior for both $L_{p}$ and *N*, adequately follows the trend of sound level. The greater the interval in time averages, the peaks of short event high noises are smoothed, obtaining a better prediction and adjustment of the model comparing with models of shorten intervals.

**Figure 4:**
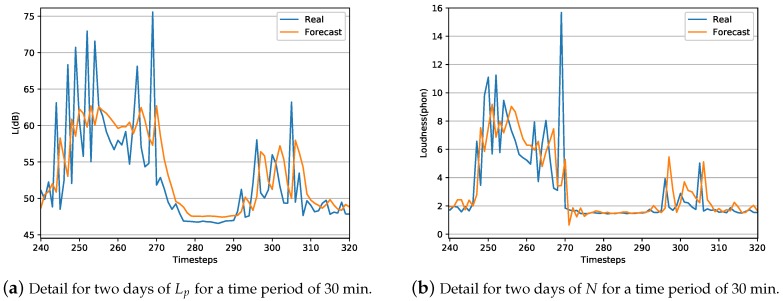
Detailed representation for two days, Friday and Saturday, for a time period of 30 min of captured and estimated LSTM data.

In order to explore in detail the obtained LSTM models, Figure 4a shows a zoomed view of graph of Figure 2d and Figure 4b shows a zoomed view of graph of Figure 3d for a two days interval with a time average of 30 min. It can be observed that the LSTM model has difficulties in precisely estimate short-time events where the sound level increase and decrease drastically, i.e., when sound level suddenly rise or decay. However, the behavior of the LSTM model is much more stable when the peaks are less relevant, e.g., during Saturdays.

### 4.2. Assessing the Robustness of the Proposed LSTM Model

In the previous section, it was concluded that the LSTM technique can develop precise models for predicting the sound parameters L${}_{p}$ and *N* in short-term. In this section, a validation of the behavior, the stability and the robustness of the LSTM technique is carried out throughout different types of tests. The objective is to analyze the variability of the LSTM models when a greater amount of samples are predicted having a smaller amount of training samples. The validations that have been made are as follows:

- 80% train and 20% test (80/20)—approximately 40 days to train and 10 days to test (validation already done in the previous experiment, used to analyze and compare).
- 70% train and 30% test (70/30)—approximately 35 days to train and 15 days to test.
- 60% train and 40% test (60/40)—approximately 30 days to train and 20 days to test.

Table 5 shows the values of RMSE, MAE, PCC and $R^{2}$ of the validations indicated for noise60 and noise30 data-sets. Analyzing the results for the parameter L${}_{p}$, it can be appreciated how independently of the type of validation the RMSE error is, around 4 dB for the noise60 data-set and around 4.3 dB for the noise30 data-set. The variations of the LSTM models for both data-sets are minimal when the type of validation performed is changed. These minimum variations can be seen with the value of $R^{2}$ that hardly suffers variations of 0.04 points. Regarding the *N* parameter, the results are very similar to the $L_{p}$ parameter in terms of model variability. Analyzing the RMSE value of the *N* parameter, it is observed that it is around 2 dB for any of the two data-sets and any of the validations. The same happens with the determination coefficient $R^{2}$ where the differences between models of different validations and data-sets do not exceed 0.05 points. A remarkable aspect of the *N* parameter for the 60/40 validation is that it gets the best result than the other validations for both the noise30 and noise60 data-sets. The explanation for this situation can be that by obtaining more test days, these days include more weekends where the noise is more stable and there are fewer punctual peaks, hence the model fit is better.

After detailing and analyzing the results of the various performed validations together with the comparison with the ARIMA technique in the previous experiment, it can be concluded that the LSTM technique obtains a considerably stable and satisfactory performance for the problem posed. It must be taken into account that the challenges presented by the LSTM technique have allowed us to make reliable models regarding the error and the adjustment of the model using very few training samples and allowing a prediction of 20 consecutive days. Although the LSTM models created follow the trend of sound with a stable behavior, they present limitations in detecting impulsive short events, i.e., high peak noises at certain times.

## 5. Conclusions and Future Work

Wireless acoustic sensor networks are an important tool for monitoring and managing noise pollution in cities. In addition to economic cost savings as compared to traditional procedure to create a noise map, these networks are helping in the design of new noise maps with extended sound sources information and enabling existing noise maps to be updated dynamically. However, it must be taken into account that sensors within a network can fail or that network signal coverage may drop in certain situations, producing missing values in the IoT platform. Moreover, it would be helpful for local administrations to know in advance the trend in noise levels in cities in the temporal short-term. As a support to address these issues and even to decrease the number of necessary nodes in a network, the techniques of artificial intelligence can help through the execution of its different algorithms.

This paper proposes the use of a deep neural network, specifically a Long Short-Term Memory neural network (LSTM) to forecast future time values creating a model that represents the behavior of an acoustic environment in a certain location, specifically sound pressure level ($L_{p}$) and loudness values (*N*) parameter are contemplated. To create this model, values taken from a node of a deployed acoustic sensor network that collects information every minute have been used. Different models have been designed for $L_{p}$ and *N* applying several time periods varied up to 60 min, in order to assess and analyze the behavior of the acoustic environment at different time intervals. To validate the model, it has been compared with the Auto Regressive Integrated Moving Average (ARIMA) time series technique, to evaluate and discuss the benefits and limitations of the proposed LSTM. Besides, to analyze the stability of the LSTM technique, several types of validations have been made. The results indicate that LSTM models obtain a lower prediction error and a better model fit than ARIMA. In general, the results achieved through the application of the LSTM technique are satisfactory since all the created models predict in a correct way the rising and falling trends of the sound levels. Moreover, obtained root mean square error values are lower than 4.3 dB for $L_{p}$ and lower than 2 phons for *N* all considered models. Analyzing the parameters separately, using the *N* level more robust models than $L_{p}$ are obtained, resulting in smaller error values and no significance differences between considered time periods. Regarding the $L_{p}$ level models, a more reliable model is achieved when a higher time period is considered. Although $L_{p}$ is a parameter with higher variance than *N*, the trend of the behavior pattern estimated by the model is satisfactory in terms of determination coefficient. Regarding the results of the different validations, these indicate that the proposed LSTM technique has little variability and needs little training data to obtain good predictions, therefore, the technique could be applied in any city, without the need to obtain long previous historical data. Regarding the limitations of the proposed LSTM technique, the difficulty of the model to follow the trend of high sound levels of the L${}_{p}$ and *N* parameters has been observed.

As a future work, an evaluation of the implementation of LSTM models within the nodes of the network of acoustic sensors is proposed. Moreover, a study to determine the influence of other climatic parameters or variables in predicting acoustic pollution through a multivariate neural network is of interest.

## Figures and Tables

**Table 1:** Number of samples per data-set for each of the pressure level and loudness parameters.

Data-Sets	Total Instances L${}_{p}$	Total Instances *N*
**noise01**	72,300	72,300
**noise05**	14,460	14,460
**noise15**	4820	4820
**noise30**	2410	2410
**noise60**	1205	1205

**Table 2:** Optimal parameters for LSTM execution experiments.

Parameter	Value
Number of input neurons	$L_{p}$[50:100]
	*N*[17:70]
Batch size	32
Number of epochs	100
Learning factor	0.001
Optimizer	Adam
Activation function	hyperbolic tangent
Loss Function	quadratic mean error
Delay Sequence	6

**Table 3:** Representation of Root Mean Square Error (RMSE), Mean Absolute Error (MAE), Pearson Correlation Coefficient (PCC) and $R^{2}$ of the five data-sets of sound pressure level values ($L_{p}$) for the LSTM proposed models and the ARIMA models.

	$L_{p}$-noise60		$L_{p}$-noise30		$L_{p}$-noise15		$L_{p}$-noise05		$L_{p}$-noise01	
	ARIMA	LSTM	ARIMA	LSTM	ARIMA	LSTM	ARIMA	LSTM	ARIMA	LSTM
RMSE	9.3000	3.9400	112.0704	4.2700	9.5734	4.2500	78.2656	3.9500	6.0694	3.5900
MAE	6.6400	2.7500	2.2050	2.8500	7.7755	2.5500	5.8934	2.0700	4.4851	1.7100
PCC	0.1732	0.8600	0.0131	0.8300	0.2798	0.8100	0.0335	0.8100	0.0521	0.8000
$R^{2}$	0.0300	0.7500	0.0002	0.6900	0.0783	0.6600	0.0011	0.6400	0.0027	0.5800

**Table 4:** Representation of RMSE, MAE, PCC and $R^{2}$ of the five data-sets of loudness values (*N*) for the LSTM proposed models and the ARIMA models.

	*N*-noise60		*N*-noise30		*N*-noise15		$L_{p}$-noise05		*N*-noise01	
	ARIMA	LSTM	ARIMA	LSTM	ARIMA	LSTM	ARIMA	LSTM	ARIMA	LSTM
RMSE	3.1100	1.9900	14.6412	2.0100	8.4290	1.9600	12.3481	1.8700	3.1400	1.7900
MAE	2.7100	0.9900	2.2050	1.0500	2.0675	1.0000	1.8617	0.8900	0.1563	0.7400
PCC	0.2000	0.7900	0.0198	0.7800	0.3769	0.7800	0.0031	0.7800	0.0011	0.7600
$R^{2}$	0.0400	0.6000	0.0004	0.5900	0.1420	0.6000	0.0000	0.6100	0.0000	0.5700

**Table 5:** Representation of RMSE, MAE, PCC and $R^{2}$ for different training and test percentages of $L_{p}$ and *N* values.

	Sound Pressure Level				Loudness				
**Data-Set**	**Train/Test**	**RMSE**	**MAE**	**PCC**	$R^{2}$	**RMSE**	**MAE**	**PCC**	$R^{2}$
**noise30**	**80/20**	4.27	2.85	0.83	0.69	2.01	1.05	0.78	0.59
	**70/30**	4.32	2.74	0.82	0.68	2.08	1.09	0.77	0.59
	**60/40**	4.51	3.15	0.84	0.65	2.00	1.05	0.80	0.63
**noise60**	**80/20**	3.94	2.75	0.86	0.75	1.99	0.99	0.79	0.60
	**70/30**	4.13	2.92	0.85	0.72	2.03	1.17	0.79	0.61
	**60/40**	4.05	3.13	0.86	0.74	1.97	1.14	0.82	0.65

## References

[1] European Commission (2002). END, Directive 2002/49/EC of the European Parliament and of the Council of 25 June 2002 relating to the Assessment and Management of Environmental Noise.

[2] Hornikx M. (2016). Ten questions concerning computational urban acoustics. Build. Environ..

[3] Murphy E., King E.A. (2010). Strategic environmental noise mapping: Methodological issues concerning the implementation of the EU Environmental Noise Directive and their policy implications. Environ. Int..

[4] Murphy E., Rice H.J., Meskell C. Environmental noise prediction, noise mapping and GIS integration: The case of inner Dublin, Ireland. Proceedings of the 8th International Transport Noise and Vibration Symposium.

[5] Arana M., Martín R.S., Martin M.L.S., Aramendía E. (2010). Strategic noise map of a major road carried out with two environmental prediction software packages. Environ. Monit. Assess..

[6] Garg N., Maji S. (2014). A critical review of principal traffic noise models: Strategies and implications. Environ. Impact Assess. Rev..

[7] Steele C. (2001). A critical review of some traffic noise prediction models. Appl. Acoust..

[8] Guarnaccia C. (2013). Advanced tools for traffic noise modelling and prediction. WSEAS Trans. Syst..

[9] Barry T., Reagan J.A. (1978). FHWA Highway Traffic Noise Prediction Model.

[10] Li B., Tao S., Dawson R.W., Cao J., Lam K. (2002). A GIS based road traffic noise prediction model. Appl. Acoust..

[11] Van Leeuwen H.J.A. (2000). Railway noise prediction models: A comparison. J. Sound Vib..

[12] Lui W.K., Li K.M., Ng P.L., Frommer G. (2006). A comparative study of different numerical models for predicting train noise in high-rise cities. Appl. Acoust..

[13] Van Leeuwen H.J.A. (1996). Noise Predictions Models to Determine the Effect of Barriers Placed Alongside Railway Lines. J. Sound Vib..

[14] Oerlemans S., Schepers J.G. (2009). Prediction of wind turbine noise and validation against experiment. Int. J. Aeroacoust..

[15] Tadamasa A., Zangeneh M. (2011). Numerical prediction of wind turbine noise. Renew. Energy.

[16] Maisonneuve M., Stevens M., Ochab B. (2010). Participatory noise pollution monitoring using mobile phones. Inf. Polity.

[17] Akyildiz I., Su W., Sankarasubramaniam Y., Cayirci E. (2002). Wireless sensor networks: A survey. Comput. Netw..

[18] Peckens C., Porter C., Rink T. (2018). Wireless sensor networks for long-term monitoring of urban noise. Sensors.

[19] Basten T., Wessels P. An overview of sensor networks for environmental noise monitoring. Proceedings of the 21st International Congress on Sound and Vibration (ICSV21).

[20] Alías F., Alsina-Pagés R. (2019). Review of Wireless Acoustic Sensor Networks for Environmental Noise Monitoring in Smart Cities. J. Sens..

[21] Mydlarz C., Salamon J., Bello J.P. (2017). The implementation of low-cost urban acoustic monitoring devices. Appl. Acoust..

[22] Camps-Farrés J. Barcelona noise monitoring network. Proceedings of the EuroNoise 2015.

[23] Bartalucci C., Borchi F., Carfagni M., Furferi R., Governi L. Design of a prototype of a smart noise monitoring system. Proceedings of the 24th International Congress on Sound and Vibration (ICSV24).

[24] Mietlicki C., Mietlicki F. Medusa, a new approach for noise management and control in urban environment. Proceedings of the 11th European Congress and Exposition on Noise Control Engineering (Euronoise2018).

[25] Navarro J.M., Tomas-Gabarron J.B., Escolano J. (2017). A big data framework for urban noise analysis and management in smart cities. Acta Acust. United Acust..

[26] Langkvist M., Karlsson L., Loutfi A. (2014). A review of unsupervised feature learning and deep learning for time-series modeling. Pattern Recognit. Lett..

[27] Che Z., Purushotham S., Cho K., Sontag D., Liu Y. (2018). Recurrent neural networks for multivariate time series with missing values. Sci. Rep..

[28] Wittenburg G., Dziengel N., Wartenburger C., Schiller J. A system for distributed event detection in wireless sensor networks. Proceedings of the 9th ACM/IEEE International Conference on Information Processing in Sensor Networks (IPSN’10).

[29] Kim H.G., Kim J.Y. (2017). Environmental sound event detection in wireless acoustic sensor networks for home telemonitoring. China Commun..

[30] Luque A., Romero-Lemos J., Carrasco A., Barbancho J. (2018). Improving Classification Algorithms by Considering Score Series in Wireless Acoustic Sensor Networks. Sensors.

[31] Zhang Y., Fu Y., Wang R. (2018). Collaborative representation based classification for vehicle recognition in acoustic sensor networks. J. Comput. Methods Sci. Eng..

[32] Cobos M., Perez-Solano J.J., Felici-Castell S., Segura J., Navarro J.M. (2014). Cumulative-sum-based localization of sound events in low-cost wireless acoustic sensor networks. IEEE/ACM Trans. Audio Speech Lang. Process..

[33] Sevillano X., Socoró J.C., Alías F., Bellucci P., Peruzzi L., Radaelli S., Benocci R. (2016). DYNAMAP— Development of low cost sensors networks for real time noise mapping. Noise Mapp..

[34] Segura-Garcia J., Navarro-Ruiz J., Perez-Solano J., Montoya-Belmonte J., Felici-Castell S., Cobos M. (2018). Torres-Aranda, Spatio-Temporal Analysis of Urban Acoustic Environments with Binaural Psycho-Acoustical Considerations for IoT-Based Applications. Sensors.

[35] Bello J.P., Silva C., Nov O., Dubois R.L., Arora A., Salamon J., Doraiswamy H. (2019). SONYC: A system for monitoring, analyzing, and mitigating urban noise pollution. Commun. ACM.

[36] Jakob A., Marco G., Stephanie K., Robert G., Christian K., Tobias C., Hanna L. A Distributed Sensor Network for Monitoring Noise Level and Noise Sources in Urban Environments. Proceedings of the 2018 IEEE 6th International Conference on Future Internet of Things and Cloud (FiCloud).

[37] Socoró J., Alías F., Alsina-Pagès R. (2017). An anomalous noise events detector for dynamic road traffic noise mapping in real-life urban and suburban environments. Sensors.

[38] Li Y., Liu M., Drosos K., Virtanen T. (2019). Sound event detection via dilated convolutional recurrent neural networks. arXiv.

[39] Yu L., Kang J. (2009). Modeling subjective evaluation of soundscape quality in urban open spaces: An artificial neural network approach. J. Acoust. Soc. Am..

[40] Lopez-Ballester J., Pastor-Aparicio A., Segura-Garcia J., Felici-Castell S., Cobos M. (2019). Computation of Psycho-Acoustic Annoyance Using Deep Neural Networks. Appl. Sci..

[41] Mansourkhaki A., Berangi M., Haghiri M., Haghani M. (2018). A neural network noise prediction model for Tehran urban roads. J. Environ. Eng. Landsc. Manag..

[42] Pedersen K., Transtrum M.K., Gee K.L., Butler B.A., James M.M., Salton A.R. (2018). Machine learning-based ensemble model predictions of outdoor ambient sound levels. Proc. Meet. Acoust..

[43] Torija A.J., Ruiz D.P., Ramos-Ridao A.F. (2012). Use of back-propagation neural networks to predict both level and temporal-spectral composition of sound pressure in urban sound environments. Build. Environ..

[44] Garg N., Soni K., Saxena T.K., Maji S. (2015). Applications of Autoregressive integrated moving average (ARIMA) approach in time-series prediction of traffic noise pollution. Noise Control. Eng. J..

[45] Tong W., Li L., Zhou X., Hamilton A., Zhang K. (2019). Deep learning PM 2.5 concentrations with bidirectional LSTM RNN. Air Qual. Atmos. Health.

[46] Krishan M., Jha S., Das J., Singh A., Goyal M.K., Sekar C. (2019). Air quality modelling using long short-term memory (LSTM) over NCT-Delhi, India. Air Qual. Atmos. Health.

[47] Noriega-Linares J.E., Rodriguez-Mayol A., Cobos M., Segura-Garcia J., Felici-Castell S., Navarro J.M. (2017). A Wireless Acoustic Array System for Binaural Loudness Evaluation in Cities. IEEE Sens. J..

[48] Raspberry PI. https://www.raspberrypi.org.

[49] Zwicker E., Fastl H. (2013). Psychoacoustics: Facts and Models.

[50] Hochreiter S., Schmidhuber J. LSTM can solve hard long time lag problems. Proceedings of the 9th International Conference on Neural Information Processing Systems.

[51] Brockwell P.J., Davis R.A. (2016). Introduction to Time Series and Forecasting.

[52] Box G.E., Jenkins G.M. (1976). Time Series Analysis. Forecasting and Control.

[53] Legates D.R., McCabe G.J. (1999). Evaluating the use of “goodness-of-fit” measures in hydrologic and hydroclimatic model validation. Water Resourc. Res..

